# Analysis of the Relationship between Economic Development and Water Resources–Ecological Management Capacity in China Based on Nighttime Lighting Data

**DOI:** 10.3390/ijerph20031818

**Published:** 2023-01-19

**Authors:** Mengjiao Wang, Xiaofang Xu, Liyuan Zheng, Xiaolu Xu, Yukuo Zhang

**Affiliations:** 1 Business School, Hohai University, Nanjing 211100, China; 2 School of Management, Wenzhou Business College, Wenzhou 325035, China; 3 School of Finance and Taxation, Zhengzhou Technology and Business University, Zhengzhou 451400, China

**Keywords:** economic development, water resources–ecological management capacity, coupling coordination relation, night light data, space effect

## Abstract

Water resources are important factors limiting social and economic development, so how to ensure the coordination between economic development and water resources–ecological management capacity has become a key issue that needs to be addressed urgently for China’s high-quality economic development. This paper used nighttime light data as proxy variables of economic development to calculate the coupling coordination degree between provincial economic development and water resources–ecological management capacity in China from 2004 to 2019 based on the coupling coordination degree model; w constructed a spatial econometric model to explore the spatial correlation and influencing factors between economic development and water resources–ecological management capacity. The results are shown in the following: (1) The overall level of China’s economic development is on an upward trend, but the regional development is unbalanced, showing a decreasing spatial pattern distribution of the eastern coastal region–mid-western region–far-western region. (2) The level of water resources–ecological management capacity is low, and the spatial distribution shows a decreasing trend in the far west–central and western–eastern coastal regions. (3) The level of coupling and coordination between economic development and water resources–ecological management capacity rises from a mild imbalance level to a little imbalance level, and the spatial distribution is consistent with the spatial distribution of economic development. (4) The factors influencing the level of coupling and coordination of inter-provincial economic development and water resources–ecological management capacity in China mainly involve the population scale, technological progress, affluence, and foreign direct investment. Each province and city should take into account its own actual situation and develop targeted measures to promote the coordinated development of economic development and the water resources–ecological management capacity.

## 1. Introduction

**Figure 1:**
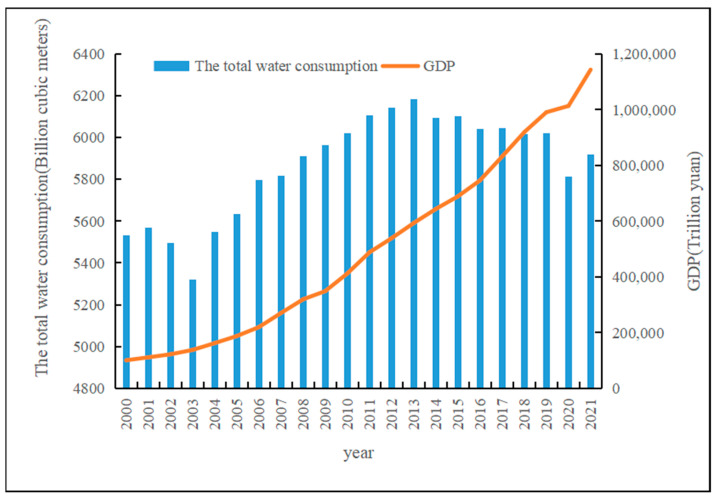
The temporal trends of total water consumption and GDP.

As a basic resource, water resources not only play an important role in the ecological environment but also a pivotal role in the national economy and society. Although China is rich in water resources, its per capita water resources only account for 25% of the world’s per capita water resources, and China is identified as a “water scarcity country” by the United Nations. With the rapid development of the social economy and the advancement in urbanization, the demand for water resources is growing in human society. China’s total water consumption increased from 550 billion cubic meters in the early 21st century to 600 billion cubic meters at present, as shown in Figure 1. Water resources have become restrictive factors for the sustainable development of China’s economy. In the early stage of reform and opening up, China’s economic development was dominated by energy-intensive and extensive industrial development at the expense of large amounts of water resources [1]. The high consumption of water resources caused by rapid economic development made China’s water environment face great challenges in pollution discharge and over-exploitation of water resources. At present, China’s economy has entered a stage of high-quality development, in which the research on the matching of resources, environment, and economic development is particularly important, especially the basic resources led by water resources. As a carrier of material circulation, water resources, ideally, should form a dynamic balance state with economic development. On the premise of not damaging the local society and ecosystem, water resources in this region have a certain carrying capacity for the three major industries, ecological environment, and city scale. With the improvement in the economic development quality, the utilization efficiency of water resources should also be improved accordingly [2]. The effectiveness of socioeconomic development strategies largely depends on the adequate supply of basic resources, and water resources play a key role in economic development strategies [3].

Scholars have conducted extensive studies on the relationship between economic development and water resources. Some studies tested the effects of water consumption on economic growth in sub-Saharan Africa (SSA) countries by using the super-logarithmic production model, and the results showed that water resources and labor were the main driving forces of economic growth in these countries [4]. Howe [5] qualitatively discussed the static relationship between water resource utilization and economic growth and believed that reasonable water resource utilization was conducive to economic growth. From the perspective of sustainable development, Bithas [6] analyzed the important role of improving the utilization efficiency of water resources on economic sustainability. Hao [7] studied the role of water resources in China’s economic development by using a simultaneous equation model, and the results indicated that industrial water has a positive contribution to economic development. Zhang et al. [8] studied the relationship between economic development and water use by using the water footprint theory, and the results showed that the economic development and water use in northwest China had not yet reached the decoupling state. Qi et al. [9] constructed a comprehensive index of the socioeconomic consumption level of regional water resources and determined the inflection point of water resources restricting economic development by using the ratio of water use change to unit GDP growth. Some scholars used the environmental Kuznets hypothesis to study the impact of economic development on water resources. They argued that economic growth to a certain extent was likely to reduce water demand [10,11,12], which was in line with the EKC hypothesis; that is, the relationship between ecological environment and economic development is inverted U-shaped. Some scholars denied the validity of EKC’s conclusion [13], and they believed that economic development and water resources showed other shapes, such as the monotonical rising-type and N-type [1]. Xu et al. [14] showed that economic development plays a role in water resources, and improvements in the water resource utilization efficiency and water environment benefit from the financial and technical support brought by economic development. Zhang et al. [15] studied the spatiotemporal distribution of the coupling coordination between water resources and economic–social systems in Guizhou Province by constructing evaluation indexes of water resource systems and economic–social systems by using the coupling coordination model; the results showed that the coordination level was generally low. Wang et al. [16] used the Lotka–Volterra model to analyze the synergistic relationship between urbanization and water use efficiency in China; the results showed that the distribution characteristics of the urbanization level and water use efficiency in China were inconsistent, and the coordination levels of the two were relatively low in mainland China and needed to be improved. Overall, the current research progresses on the relationship between water resources and economic development focused on one-way effects, namely the impacts on the economic development of water resources or the effects of economic development on the water resource system, and less research on the interaction between both, on the regional scale in China, and the time and space distributions of the water resource system and economy coordinated development research.

Current studies use water resource carrying capacity, water resource utilization efficiency, and other indicators to evaluate water resource system Some scholars used the catastrophe progression method to quantify the evaluation indicators, to evaluate the provincial water resource carrying capacity in China [17,18]. Wang et al. [19] constructed an evaluation index system of water resource carrying capacity in Hebei Province by using the principal component analysis method. Cao et al. [20] established an evaluation system by using the entropy method to determine the index weight and evaluate the water resource carrying capacity in Hunan Province. Some scholars have used the system dynamics method to evaluate the carrying capacity of water resources [21,22]. Zhao et al. [23] evaluated the carrying capacity of land–water resources in Central Asia by using ecological and water footprint methods. Wang et al. [24] used the super-efficiency DEA (data envelopment analysis) model to measure the water resource utilization efficiency of 30 provinces in China. Zhao et al. [25] measured the water resource utilization efficiency of 31 provincial administrative regions in China from 2001 to 2014 and the efficiency of each stage by adopting a slack-based efficiency evaluation model based on the relaxation of two stages to consider undesirable output. In this study, water resources–ecological management capacity was used to evaluate the water resource systems. The water resources–ecological management capacity was measured by constructing a water resources–ecological management index.

It is worth noting that previous studies have mostly measured economic development using traditional economic statistics related to GDP [26,27], and such economic indicator data may have collection or calculation errors. At the same time, because the performance of Chinese officials is tied to GDP, local government officials may be promoted by falsifying GDP data [28]. As a result, the use of statistics such as GDP to measure economic development has been questioned by outsiders [29]. In modern society, all economic activities survive in nighttime performances, and light is the dominant information for nighttime performances; the more intense the economic activity, the more intense the nighttime light will be. Therefore, compared with GDP statistics, nighttime lighting data eliminate the maximum artificial factors and are more objective. Scholars have shown that nighttime light observation data can more accurately reveal the level of local economic development [30]. Martinez, Luis Roberto [31] used night light data to examine the differences in how different polities exaggerate their economic growth. They found that GDP and nighttime light data were both positively correlated with economic growth in different countries, but the credibility of the two differed significantly; GDP data were more susceptible to government manipulation and nighttime light data (based on satellite observations being more objective). By analyzing the calibrated nighttime light data, it was found that there is a strong correlation between nighttime light density and local economic development in Chinese regions [32].

The coupled coordination theory can better make a comprehensive evaluation of two or more systems and can be used to analyze the degree of interaction and mutual influence between systems. Existing studies have applied the coupled coordination theory to water-energy–food systems [33], soil and plant systems [34], urbanization development and ecosystems [35], and other fields, while the current situation of coupled coordination between water resources–ecological management capacity and economic development has been less studied. Studying the coupling and coordination relationship between water resources–ecological management capacity and economic development is of great significance to promote high-quality economic development under the new normal.

Therefore, this paper innovatively used nighttime lighting data as proxy variables for economic development and a coupled coordination model to analyze the spatial and temporal evolutions and spatial distribution differences of the coupled coordination degree of economic development and water resources–ecological management capacity in 31 provinces of China from 2004 to 2019. Then we used a spatial panel model to conduct an exploratory analysis of the spatial characteristics and influencing factors of the coordination degrees of the two, to provide a reference for the sustainable development of economic and water resources. Compared with existing studies, the contributions of this paper are as follows: (1) the use of nighttime lighting data as proxy variables for economic development avoid the subjectivity of statistical indicators and make the research conclusions more convincing; (2) existing studies mostly focus on the one-way influence of the relationship between economic development and water resources, but rarely focus on the mutual influence of the two. This study uses a coupled coordination model to analyze the reciprocal feedback between economic development and water resources–ecological management capacity to provide implications for the sustainable development of economic development and the ecological environment, especially the water resources environment. (3) The existing studies on economic development and water resources environments are mostly limited to the assessment of the current situation, and few have explored the spatial effects of the coupled and coordinated relationship between the two under regional heterogeneity. This paper analyzes the spatial effects of the coordination between economic development and water resources–ecological management capacity based on the study of coordination between the two and explores the influencing factors that affect the spatial effects of both.

The rest of this paper is organized as follows. The second section presents the evaluation method and data sources. The third section presents the analysis of the results. The fourth section presents the analysis of the factors influencing the coordination of economic development and water resources–ecological management capacity. The fifth section presents the summary of the whole paper and policy recommendations.

## 2. Research Methodology and Data Sources

### 2.1. Measurement of Regional Economic Development Based on Nighttime Light Data

Nighttime light data have the characteristics of strong spatial directionality, easy access to information, and timeliness. The existing studies show that nighttime lighting is highly consistent with the distribution of economic activities [36], so this paper selected nighttime lighting density as a proxy variable for the regional economic development index [37,38] to measure the regional economic development level of each province in China from 2004 to 2020. High-brightness areas have obvious urban agglomeration effects and high socioeconomic development, while low-brightness areas have relatively weak economic development. Currently, global nighttime light data are mainly obtained from the DMSP/OLS nighttime light data of the U.S. Defense Meteorological Satellite Program (DMSP) and the NPP/VIIRS data from the new generation of Earth observation satellites launched by the U.S. The DMSP/OLS nighttime light data cover the years 1992–2013 and the NPP/VIIRS nighttime light data cover the years 2012–2020.

### 2.2. Measurement of Water Resources–Ecological Management Capacity

Water resources–ecological management capacity covers factors, such as water resources endowment, economic and technological development, socioeconomic system support situations, ecological and environmental protection, etc. This paper refers to the study by Zheng et al. (2021) to construct the water resources–ecological management index and classify the index into four subsystems: water resource system, water social system, water economic system, and water ecological environment from the perspective of supply and demand; we constructed the index system as shown in Table 1. We used the entropy weight method to calculate the weights and finally derived the water resources–ecological management index value.

In this paper, the entropy value method was selected to determine the index weights, and then the standardized value of the index was multiplied by the value of the weight to measure the water resources–ecological management index of a region. The value of the water resources–ecological management index is between 0 and 1. Close to 1 indicates strong water resources–ecological management capacity, and close to 0 indicates weak water resources–ecological management capacity. The specific steps are as follows.

(1) Standardization of indicators.

(1) $$y_{ij}=\{\begin{array}{c} \frac{\left(x_{ij}-\min x_{j}\right)}{\left(\max x_{j}-\min x_{j}\right)},x_{j}\;\mathrm{is}\;\mathrm{positive}\;\mathrm{indicator}\\ \frac{\left(\max x_{j}-x_{ij}\right)}{\left(\max x_{j}-\min x_{j}\right)},x_{j}\;\mathrm{is}\;\mathrm{negative}\;\mathrm{indicator} \end{array}\;$$

In Formula (1), $x_{ij}$ indicates the value of the *j*th indicator of the *i*th province of the water resources–ecological management capacity, $y_{ij}$ in Formula (1) indicates the standardized value of the *j*th indicator of the *i*th province, and if the standardized value calculated by Formula (1) is equal to zero, it will be recorded as 0.00001.

(2) We calculate the weight of each indicator using the entropy value method. The entropy value of indicator *j* and the weight of indicator *j* are calculated as follows.

(2) $$e_{j}=-\frac{1}{lnm}\overset{m}{\underset{i=1}{\sum }}\frac{y_{ij}}{\sum_{i=1}^{m}y_{ij}},$$

(3) $$\;w_{j}=\frac{1-e_{j}}{\sum_{j=1}^{n}\left(1-e_{j}\right)}\;$$

In Formula (2), $e_{j}$ indicates the entropy value of the indicator *j*; in Formula (3), $w_{j}$ indicates the weight of indicator *j*.

(3) Water resources–ecological management index measurement.

(4) $$S_{i}=\overset{m}{\underset{j=1}{\sum }}w_{j}y_{ij}\;$$

In Formula (4), $S_{i}$ indicates the value of the water resources–ecological management index of the *i*th province.

### 2.3. Coupling Coordination Model of Economic Development and Water Resources–Ecological Management Capacity

Coupling is the phenomenon where two or more systems interact and influence each other, but the system is a dynamic whole. The coupling coordination model can detect how well the coordination status between two systems is and whether it is a benign interaction. In this paper, we used the coupling coordination model to measure the degree of mutual influence and interaction between China’s economic development and water resources–ecological management capacity. The model is as follows.

(5) $$T=\alpha U_{1}+\beta U_{2}$$

(6) $$C=2\times \frac{\sqrt{\left(U_{1}\times U_{2}\right)}}{\left(U_{1}+U_{2}\right)}$$

(7) $$D=\sqrt{C\times T}$$

In the above equations, $U_{1}$ and $U_{2}$ denote economic development and the water resources–ecological management capacity, respectively, and *α* and *β* are weighting coefficients. China’s economy has gone through different stages of development. In the beginning, rapid economic growth was achieved at the expense of the environment, while water-related issues were ignored. Now that China has entered the stage of high-quality development, water resources and environmental issues are in the same important position as economic development. Therefore, this paper believes that economic development and water resources–ecological management capacity are equally important. Referring to previous studies (Chen et al., 2020; Liu et al., 2020; Li et al., 2021), we let *α* = *β* = 0.5; *C* denotes the coupling degree and varies between 0 and 1; *D* denotes the coupling coordination degree, varying between 0 and 1. The greater the *D* is, the higher the coupling coordination degree is. To better present the different development stages of the coupling coordination level, the coupling coordination degree is divided into three levels and eight stages in the study by Yang et al. [39]. The details are shown in Table 2.

### 2.4. Data Sources

Due to the availability of data, the research interval of this paper was between 2004 and 2020. The nighttime light data came from the Hong Lan data science laboratory in China. The DMSP/OLS light data and NPP/VIIRS light data had inconsistent data scales, resulting in discontinuous and incomparable data. To make DMSP/OLS nighttime light data and NPP/VIIRS data comparable, the two types of lighting data were fused by the Hong Lan data science laboratory, referring to the research by Wu et al. [40] and Li et al. [41].

**Figure 2:**
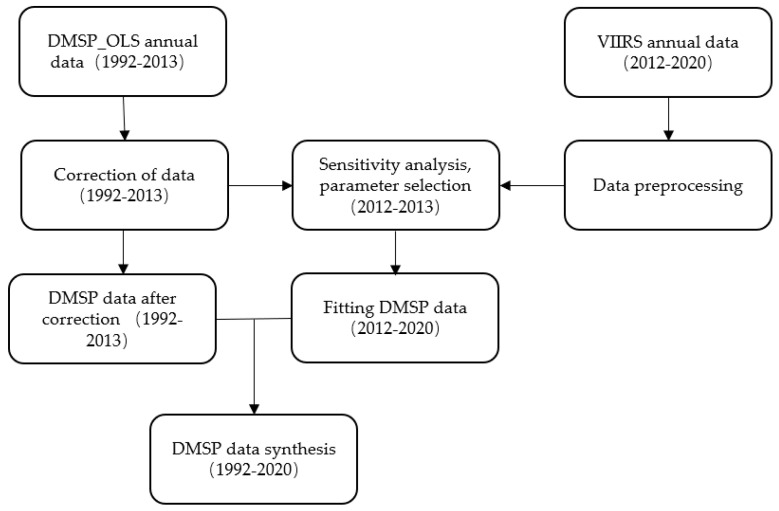
The fusion path of DMSP/OLS light and NPP/VIIRS light.

The two types of lighting data were fused as follows: (1) Data extraction. To avoid the errors formed by the image grid deformation, the influence of administrative regions on nighttime lighting was extracted and converted to the Asia_Lambert_Conformal_Conic projection coordinate system, and its spatial resolution was set to 1000 m. (2) DMSP/OLS data processing. Jixi City in Heilongjiang Province was selected as the correction point, and F182013 was used as the reference dataset because it had the highest total radiometric image value from the national perspective. The invariant objective method was used to establish a quadratic regression model to correct the DMSP/OLS data. To integrate the data captured by different satellites in the same year and eliminate the unstable sources, intra-year fusion was performed based on the corrected data, and inter-year data correction was performed on this basis to improve the continuity of the data. (3) NPP/VIIRS data processing. The data—although some preprocessing was performed on the raw satellite imaging data—did not deal with the effect of gas flares. Referring to the study by Wu et al. [40], the national cell image element radiation threshold of 472.86 was selected, and cells with negative image element values were removed. (4) We referred to the study by Li et al. [41] to convert the above NPP/VTTRS data into DMSP data. (5) We combined the DMSP data processed in the above steps with the NPP data to form continuous and comparable nighttime lighting data for each provincial and urban area in China from 1992 to 2020. Based on this paper, the nighttime lighting data of each provincial and urban area in China from 2004 to 2020 were selected as the base data for this study. The basic principle was as follows: firstly, a sensitivity analysis was performed on the data from the same year in the two sets of data and then the optimal fitting parameters were selected; secondly, according to the selected optimal parameters, the VIIRS (2012–2020) annual data were calculated and fitted to the DMSP (2012–2020) data; finally, a synthetic DMSP (1992–2020) dataset was constructed. The fusion path is shown in Figure 2 (the figure is from the Hong Lan data science laboratory).

The data from the water resources–ecological management index mainly came from China’s Statistical Yearbook and provincial statistical yearbooks. Hong Kong, Macao, and Taiwan were not considered in the study due to poor data accessibility. Individual indicators were calculated based on statistical data, and for some years with missing data, 3-year change trends were used for prediction.

## 3. Data Analysis and Results

### 3.1. Analysis of the Spatiotemporal Evolution of Economic Development and Water Resources–Ecological Management Capacity

**Figure 3:**
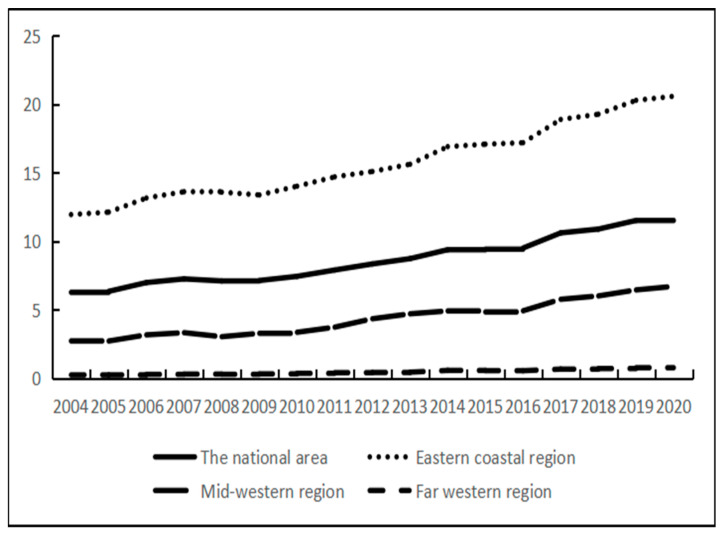
Evolution trend of the economic development level.

**Figure 4:**
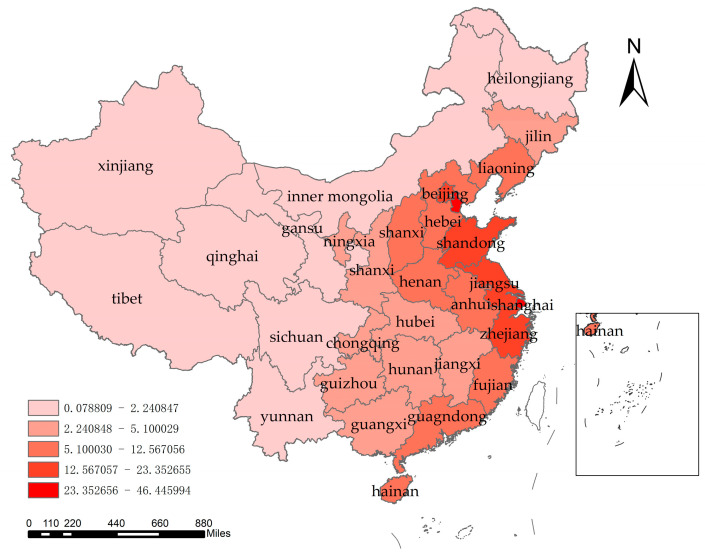
Spatial distribution of the perennial mean value of the economic development level.

The economic development index of each province in China was obtained by analyzing the nighttime lighting data of each province in China from 2004 to 2020. During the study period, China’s economic development as a whole showed an upward trend, with the average value of the economic development index increasing from 6.2612 in 2004 to 11.8287 in 2020, an increase of up to 85%. On a multi-year average, there was a decreasing change of “eastern coastal region–mid-western region–far-western region” in the space (Figure 3 and Figure 4). The economic development level was ahead of the national average in the eastern coastal region because the eastern coastal region is rich in resources and has a higher degree of openness to the outside world. The economic development index of the mid-western regions was lower than that of the whole country because of its backward openness and economic and technological levels, especially in Xizang and Xinjiang provinces. The specific change trend is shown in Figure 3. According to the changing trend in Figure 3, the economic development level of Chinese provinces can be divided into five stages in the time dimension. The first stage was from 2004 to 2006 when China’s economic development was in an upward stage from 6.26 to 6.97 due to China’s accession to WTO and the deepening of marketization. The second stage was from 2007 to 2010 when the growth rate of the economic development index slowed due to the global financial crisis. In this stage, China’s marketization level was hit. Provinces along the eastern coast, mid-western regions, and the far west were hit to varying degrees. In the third phase, from 2011 to 2014, the national economic development was in a rapid upward phase, and all regions showed the same trend. This was mainly due to the central government‘s investment of RMB 4 trillion in 2009 to sustain economic growth and expand domestic demand. This plan worked very well and boosted the economy. At the end of 2013, China’s high-speed railway “four vertical and four horizontal” trunk line pattern was formed, which accelerated the flow of production factors among regions and reshaped China’s economic landscape. The fourth stage was from 2015 to 2020 when China’s economic development entered a new stage. In the context of the new economic normal, “One Belt, One Road”, “Yangtze River Economic Belt”, “Western Development“, and other development strategy policy dividend effects began to appear, and the growth rate of economic development in the central and western regions developed more.

**Figure 5:**
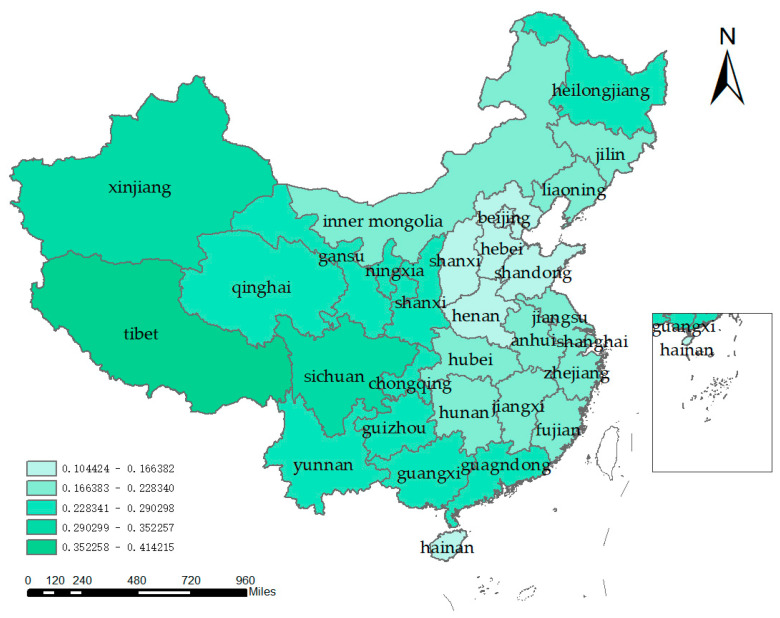
Spatial distribution of the perennial mean value of the water resources–ecological management capacity.

**Figure 6:**
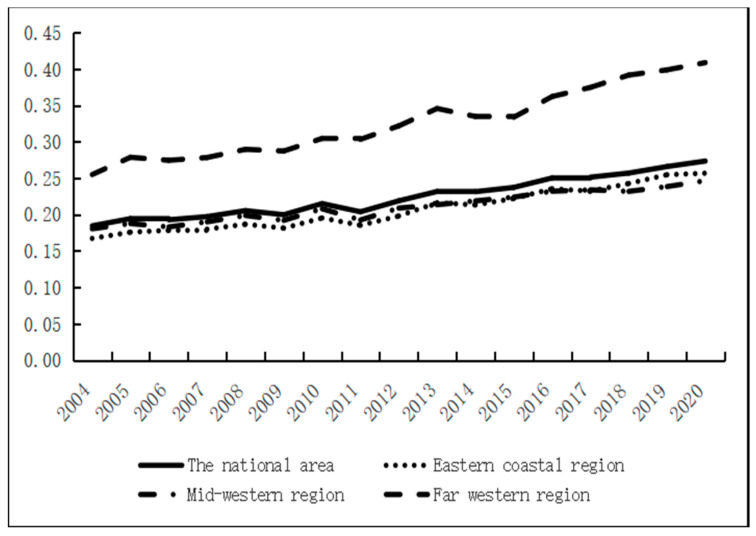
Evolution trend of the water resources–ecological management capacity.

The inter-provincial water resources–ecological management capacity level of China was calculated by constructing an index system. From the overall perspective of China, the national water resources–ecological management capacity from 2004 to 2020 increased from 0.1836 in 2004 to 0.2735 in 2020, showing a stable upward trend, but the overall water resources–ecological management capacity level needs to be improved. According to the average value (from many years), the western region, especially Tibet, has a small population, a low degree of development and utilization, and abundant water resources, leading to a high water resources–ecological management capacity. However, the population density in the eastern region is high, and the high demand for water resources makes the local water resources–ecological management capacity weak. From the north-south direction, the water resources are more in the south and less in the north, and the water resources–ecological management capacity of the northern provinces is low (Figure 5). As a whole, it shows a decreasing distribution of “far-western region–mid-western region–eastern coastal region”, as shown in Figure 6.

In the index system, the two indicators of per capita ecological water use and total water resources have greater weight, which is also the main reason for the higher water resources–ecological management capacity in the western region, mainly in Yunnan and Guizhou provinces. The rich water resources and sparse populations in the far-western region, especially in Tibetan provinces, are the main reasons for the higher water resources–ecological management capacity in the western region. The eastern coastal region shows a stable upward trend in the water resources–ecological management capacity level, and the overall mid-western region difference between the two regions is not significant. The mid-western region was slightly higher than the East Coast region from 2004 to 2015, while the East Coast region overtook the mid-western region from 2016 to 2020, but the gap is still small. In 2012, China promulgated the strictest water resource management system and established the “three red lines”, which required water use efficiency to reach or approach the world’s advanced levels by 2030. In 2015, the “Water Ten” (water pollution prevention and control action plan) was promulgated, and by the end of 2018, 31 provinces across the country had fully established the river chief system. A series of policies have been introduced to steadily improve the efficiency of water resource utilization and enhance the level of water resources–ecological management capacity.

### 3.2. Analysis of the Coupled Coordinated Development of Economic Development and Water Resources–Ecological Management Capacity

In order to eliminate the influence of the magnitude, the coupling coordination degree of economic development and water resources–ecological management capacity from 2004 to 2020 was calculated by using the coupling coordination degree model after standardizing the economic development index. From the calculation results, it can be seen that the mean value of the coupling degree between economic development and water resources–ecological management capacity in China was 0.8272, which was at a high level, indicating that the economic system and water resource system in China are closely related. In terms of time evolution, it increased from 0.75 in 2004 to 0.84 in 2020, indicating that the interaction between the two systems is increasing.

**Figure 7:**
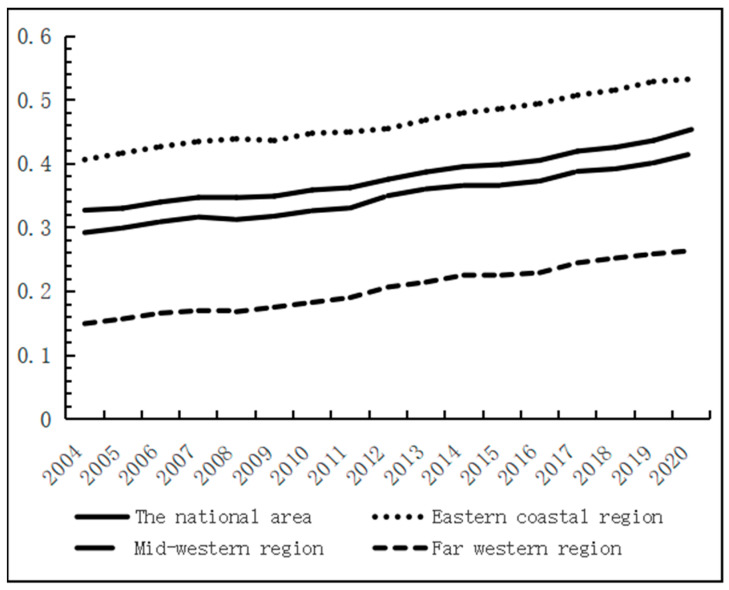
Evolution trend of the coupling coordination degrees of Chinese provinces.

The mean value of the coupling coordination degree between China’s economic development and water resource use efficiency rose steadily, as shown in Figure 7; it rose from 0.3274 in 2014 to 0.4539 in 2020. This means that the coupling coordination between China’s economic development and water resources–ecological management capacity has transitioned from a mild imbalance level of the dysfunctional recession stage to the little imbalance level of the transition stage. Compared with the coupling degree of the two systems, the coupling coordination degree is still at a low level, indicating that the economic system and the water resource system are still at the stage of mutual constraint. The lower economic level will inhibit the development of the water resources–ecological management capacity; at the same time, the low level of water resources–ecological management capacity will limit the development of the economy.

**Figure 8:**
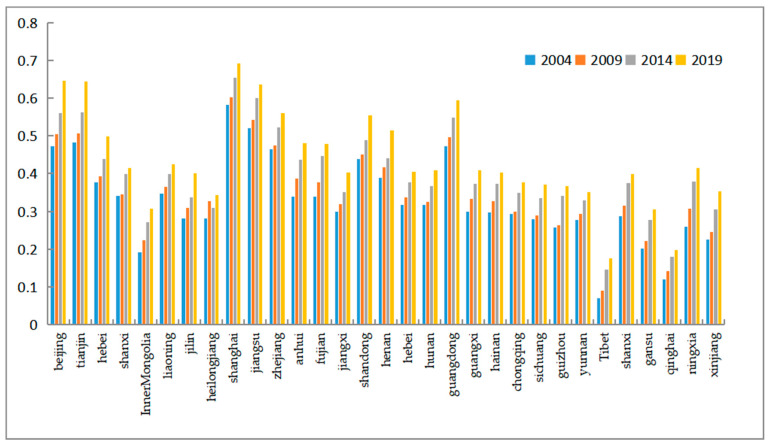
Evolution trend of the coordination level between economic development and the water resources–ecological management capacity.

**Figure 9:**
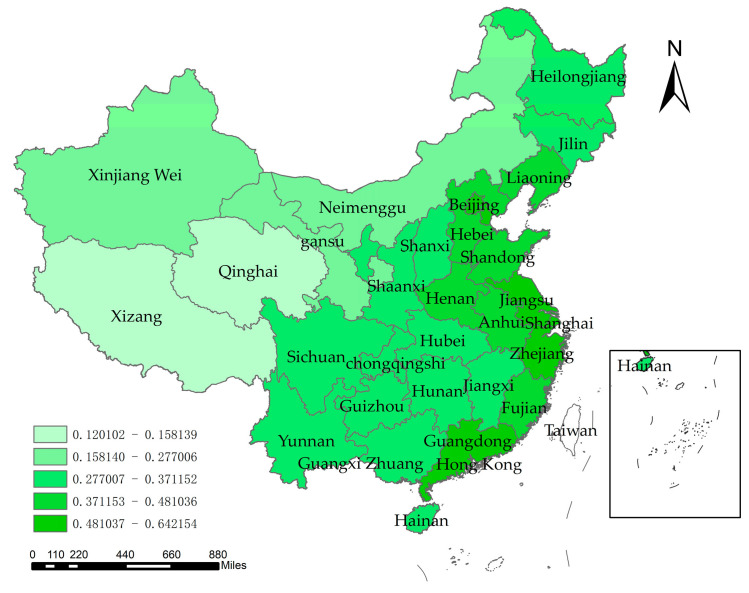
Spatial distribution of the perennial mean value of the coupling coordination level.

At the regional level, the coupling coordination of all Chinese provinces shows an increasing trend, as shown in Figure 8. The degree of coupling and coordination between economic development and water resources–ecological management capacity in China shows spatial differences, with six provinces of Beijing, Tianjin, Shanghai, Zhejiang, and Guangdong at high levels, while Tibet and Qinghai have relatively low degrees of coupling and coordination. The mean values of the coupling coordination for each province were plotted on a map of China using ArcGIS software, and the classification method was based on the infinitesimal natural breakpoint method (Figure 9). It can be seen that the coupling coordination is higher in the eastern coastal region, followed by the mid-western regions, and lowest in the far-western region. The economic development level in the far-western region is generally low and the development mode is crude. A large amount of resource consumption is used as the driving force of economic development, so although the water resources–ecological management capacity is high, the overall coupling coordination is low. From the interannual variation, as shown in Figure 7, the coupling coordination degree of each region was in an increasing trend during 2004–2020, showing a decreasing trend of “eastern coastal region–mid-western region–far-western region“. This indicates that the level of economic development dominates the interaction between the two systems. At the same time, because economically developed regions have more advanced technologies and concepts, and are more aggressive in dealing with water problems, the overall trend of coupling and coordination between the two systems is “East-Middle-West” decreasing.

The mean value of coupling coordination among the provinces in the eastern region increased from 0.4067 to 0.5329, rising from the little imbalance level in the transition phase to the basic coordination level. The mid-western region, on the other hand, increased from 0.2923 to 0.4158, rising more than the east region, from a moderate dissonance rating to an excessive stage of wear and tear. While the far-western region increased from 0.1496 to 0.2639, implying a progression from a severe to a moderate dysregulation level. In terms of growth rates, there is a decreasing trend of “far west–mid west–east coast”. This is due to the low labor costs and small industrial base in the central and far-western regions, and the gradual transfer of industries from the high development areas in the east to the west, driving local economic development, resulting in a greater increase in the coupling and coordination between economic development and water resources–ecological management capacity.

## 4. Analysis of Factors Influencing the Coupling and Coordination of Economic Development and Water Resources–Ecological Management Capacity

### 4.1. Spatial Autocorrelation Test

**Figure 10:**
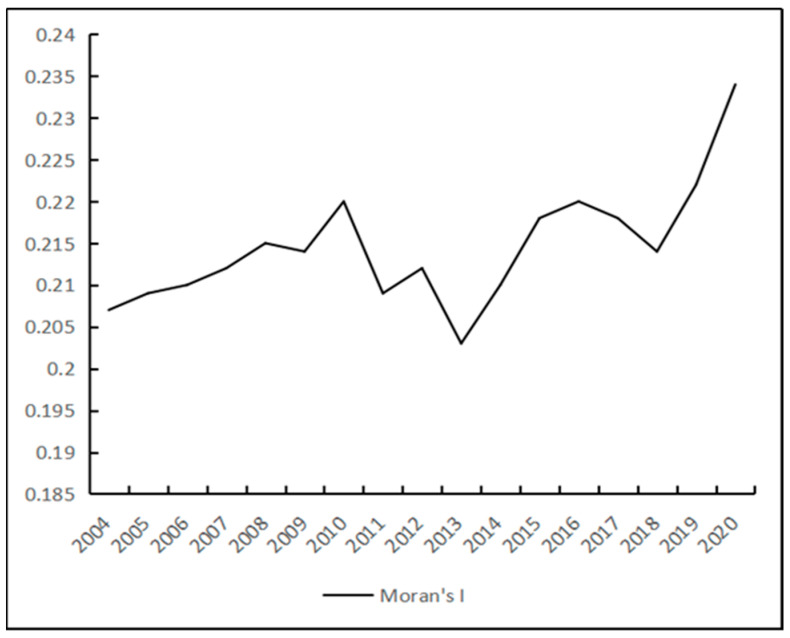
Moran index.

Based on the measured coupling coordination between economic development and water use efficiency, Moran’s I index of economic development and water use efficiency was calculated by using stata software. The spatial weight matrix was set as an asymmetric economic geographic spatial weight matrix, and the spatial dependence of the two was judged. By measuring, as shown in Figure 10, the Moran’s I index was positive in all years of the study period and passed the 10% significance level test, indicating that there was a significant positive autocorrelation between the inter-provincial economic development and the coupling coordination of the water resources–ecological management capacity in China, showing a strong spatial clustering pattern. The Moran index rose from 0.207 to 0.234, showing a weak upward trend. It showed that the spatial prominence of the coordination degree of inter-provincial economic development and water resources–ecological management capacity in China was enhanced, and the agglomeration trend was highlighted. The Moran index fluctuated strongly from 2010 to 2016, reaching the lowest point in 2013, and then it started to rebound with an upward trend, indicating that the spatial distribution pattern of the coupled coordination degree of inter-provincial economic development and water resources–ecological management capacity in China is not stable enough and is prone to change.

### 4.2. Selection of the Spatial Econometric Model

The previous study shows that there is an obvious spatial correlation and dependency between the coupling level of economic development and water resources–ecological management capacity in China. The use of ordinary regression models can underestimate or overestimate the effects of certain factors due to the influence of spatial factors, so this paper chose a spatial econometric model that could respond to spatial effects to investigate the factors influencing the degree of coordination of the two couplings. Combined with existing studies [42,43], the degree of coupled coordination between economic development and water resources–ecological management capacity is influenced by factors such as industrial structure, technological progress, affluence, foreign direct investment, and population size. The relevant factor indicators were selected as follows: industrial structure (IS) was expressed by the ratio of tertiary to secondary value added; technological progress (TEC) was measured by the number of patents granted; affluence (PGDP) was expressed by GDP per capita; foreign direct investment (FDI) was expressed by the amount of foreign direct investment; and population size (PI) was expressed by the year-end resident population.

The spatial econometric model was selected by the LM test, and the LM test was conducted on panel data of 31 provinces and cities in China from 2004 to 2020 using stata software. The test results showed that LM-error passed the significance test, while LM-lag failed. According to the Anselin criterion, the spatial error model was selected, and the asymmetric spatial weight matrix constructed above was selected as the weight matrix. The Hausman test rejected the null hypothesis at the 1% level, so this paper chose the spatial error model with fixed effects to analyze the influencing factors of the coupling coordination degree between provincial economic development and water resource utilization efficiency in China. In Table 3, the “Number of ids” represents the number of provinces in our study, which equals 31. “Observations” represents the number of IDs from 2004 to 2020, and it equals 17 times 31. “sigma2_e” means specific errors of individual effects.

According to the estimation results in Table 3, λ is significantly positive through the significance level test of 1%, indicating that there is a strong spatial clustering phenomenon in the coupled coordination of economic development and water resources–ecological management capacity in China. It confirms the need to choose a spatial model again. The coefficient of population size is 0.210, which passes the 5% significance level test, indicating that the population size contributes to the coupling and coordination of economic development and water resources–ecological management capacity. The increase in population size facilitates the accumulation of human capital and improves the level of science and technology, thus enhancing economic development. At the same time, the population concentration makes the demand for water resources increase, and the scarcity of resources forces the region to use advanced technology to improve the efficiency of water resource utilization and improve the current situation of water resources–ecological management capacity. This is the reason why the population size can positively affect the coupled level of coordination between economic development and water resources–ecological management capacity. The coefficient of technological progress was 0.003, which passed the 5% level of the significance test. Science and technology are the first productive forces, and the improvement in technological progress helps to enhance the production efficiency of enterprises and economic development, thus promoting the innovation of water resource development, utilization, and secondary use technologies [1], reducing the amount of water input per unit of output. Therefore, the efficiency of water resource utilization is improved, which improves the coupling and coordination level of economic development and water resources–ecological management capacity. The coefficient of GDP per capita is 0.032, which is significant at the 1% level. It indicates that the affluence level has a positive contribution to the coupled and coordinated level of economic development and water resources–ecological management capacity. The reason is that regions with higher GDP per capita are more economically developed and need more resources to meet their own development in the process of development, thus promoting the high-quality development of the region. The construction of ecological civilization is the focus of high-quality development, and water resources are the key elements in the construction of ecological civilization. The affluent region attaches great importance to the construction of ecological civilization so that the region can achieve rapid economic development while the water environment is improved and the water resources–ecological management capacity is increased, thus improving the coupling and coordination between the two. This conclusion was verified in the eastern coastal region of China. The coefficient of foreign direct investment is significant at the 5% level, indicating that the level of foreign direct investment positively influences the level of coupling and coordination between economic development and water resources–ecological management capacity. Foreign direct investment helps solve the capital shortage problem in economic development and brings advanced technology and management experience, generating technological spillover effects on regional enterprises. Therefore, the level of regional production technology and innovation capacity is improved, and the efficiency of resource use is increased. The eastern coastal region of China has a higher level of openness to the outside world and a higher share of foreign direct investment. However, the far-western region is deep inland and has a lower level of openness to the outside world, so the level of coupling coordination is higher in the east than in the west. The coefficient of industrial structure is −0.025 with a significance level of 5%, indicating that the industrial structure has a suppressive effect on the coupling and coordination level of economic development and water resources–ecological management capacity. During the study period, China was in the rising stage of industrialization, with well-developed heavy industries, and was in the transition period of transition from secondary to tertiary industries. However, the scale of the tertiary industry is currently limited by factors such as the layout of the population; moreover, supporting facilities have not yet formed a scale effect, limiting its function of relieving environmental pressure and improving the water resources–ecological management capacity. Therefore, it acts as a disincentive for the harmonization of economic development and water resources–ecological management capacity.

## 5. Discussion

### 5.1. The Applicability of Night Light Data for Economic Development Analysis

Using night light data as proxy variables of economic development, this study tested the coupling coordination level of economic development and water resources–ecological management capacity in 31 provinces and cities in China. This treatment is consistent with previous research. For example, Du et al. [44] studied the decoupling of economic growth and carbon emissions in 289 cities in China by using night light data as proxy variables for economic growth. Li [45] studied the spatial–temporal pattern evolution of technological innovation and regional economic development in China by using night light data as proxy variables for regional economic development.

The results of this paper show that night light data can effectively analyze the coupling coordination level of economic development and water resources–ecological management capacity. The analysis results of this study are also consistent with the results of previous studies using GDP data to measure economic development and water resource utilization. For example, the study by Zhang et al. [8] showed that China’s water resource utilization and economic development are not decoupled, and the government must control the intensity of water resources development and utilization in arid areas, which is in line with the results of this study. The results of this study show that the two systems of socio-economic development and water resources–ecological management capacity interact and influence each other.

### 5.2. Temporal Evolution of the Coupling Coordination Degree of Economic Development and Water Resources–Ecological Management Capacity

The analysis results in the previous section show that the coupling coordination of inter-provincial economic development and water resources–ecological management capacity in China has improved. During the study period, the coupling coordination level transitioned from the dysregulation recession stage to the transition stage. The improvement shows that the water management policies implemented by China’s government in recent years are effective. In recent years, in order to improve the water resources–ecological management capacity, the Chinese government has been adopting various policies to deal with the problem of water resources. In 2012, the “Strictest Water Resources Management System” was implemented to determine the double control red line of total water use and water use efficiency. In 2019, the National Water Saving Action Plan was promulgated to vigorously promote water saving throughout society and comprehensively improve the efficiency of water resource utilization. The introduction of a series of guidelines and policies shows the determination and attitude of the Chinese government in water resources management. China is still experiencing rapid urbanization, which will inevitably lead to over-exploitation and utilization of resources [46]. Although China’s economy has entered a stage of high-quality development, it is still a very big challenge to rapidly improve the coupling coordination level of water resources–ecological management capacity and economic development in a short period of time.

### 5.3. Spatial Difference of the Coupling Coordination Degree of Economic Development and Water Resources–Ecological Management Capacity

The results of this study show that the spatial distribution of the coupling coordination level of inter-provincial economic development and water resources–ecological management capacity in China is uneven. Although the water resources–ecological management capacity levels of the central and western regions are higher than those of the eastern regions, the coupling coordination levels of the eastern regions are higher than those of the central and western regions. The reason is that the advanced technology and capital of the eastern regions make the eastern regions economically developed, providing stronger concepts and abilities in water resources management [47]. Within the research range, the coupling coordination levels of the central and western regions are improved more compared to those of the eastern regions, because the national strategy of the rise of the central region and the development of the western region is making some industries transfer to the central and western regions, improving the levels of local economic development. At the same time, water resources and economic development are equally important in the coupling process. Since China’s rivers and lakes are cross-administrative regions, in order to improve the coordinated development levels of the two, it is necessary to break the restrictions of administrative regions, and provinces within the basin should unite to play a linkage effect and improve the water resources–ecological management capacity.

## 6. Conclusions 

This paper takes 31 provinces of China as the study area and uses nighttime lighting data as proxy variables for economic development, which makes up for the shortcomings of artificial intervention in socioeconomic indicators and is more objective. The integration of two types of nighttime lighting data, DMSP/OLS and NPP/VIIRS, fills the gap of DMSP/OLS nighttime lighting data after 2013 and provides data support for China’s regional economic development research with a longer time span. On this basis, the coupled coordination degree of China’s regional economic development and water resources–ecological management capacity was calculated by constructing a water resources–ecological management index system and using a coupled coordination model. Then, this paper analyzed the spatial and temporal evolution of the coupled coordination between regional economic development and water resources–ecological management capacity in China and used a spatial econometric model to explore the spatial correlation and influencing factors of economic development and water resources–ecological management capacity. Therefore, this is somewhat innovative. The results show that (1) during the study period, China’s overall economic development level was on the rise, but there was a regional imbalance, showing a spatial pattern distribution involving the eastern coastal region–mid-western region–far-western region; the water resources–ecological management capacity index was low, with a weak growth trend, and the spatial distribution showed a decreasing trend in the far-western region–mid-western region–eastern coastal region. (2) The coupling coordination between economic development and water resources–ecological management capacity increased from a mild imbalance level to a little imbalance level. At the regional level, it showed a spatial distribution pattern in the eastern coastal region–mid-western region–far-western region, which is consistent with the spatial distribution of economic development. (3) China’s inter-provincial economic development and water resources–ecological management capacity were positively spatially autocorrelated with obvious clustering trends; however, the distribution pattern was not stable enough and prone to change. Based on the regression results of the spatial error model, the population size, technological progress, affluence, and foreign direct investment level positively affect the coupled coordination level of economic development and water resources–ecological management capacity; moreover, industrial structure has a significant inhibitory effect on the coupled coordination levels of both.

The coordinated development of economic development and ecological environment is the only way to China’s high-quality economic development, and improving the water resources–ecological management capacity is an important step to protect the ecological environment. The research by Zhao et al. [48] showed that urban development and resources and environmental carrying capacity were interrelated. Whether the two can develop harmoniously is related to the fate of the city itself and whether the surrounding areas can successfully achieve the goal of high-quality development. Therefore, the coupling and coordinated development of water resources–ecological management capacity and economic development is very important for the high-quality development of the Chinese economy. To improve the level of coupling and coordination between economic development and water resources–ecological management capacity, the following recommendations are made. (1) Each local government should fully understand its own economic development and the current situation of water resources, and raise the coordination level between economic development and water resources–ecological management capacity to the strategic level. The mid-western region and far-western region should seize the opportunity of belt and road development to promote the upgrading of the industrial structure and the transformation of high water-consuming and high-polluting industries to clean industries, thus promoting the coordinated development of economic and water resources. They should pay attention to inter-regional linkage effects while allowing high-quality provinces to take the lead. The eastern region is economically developed and has a high level of science and technology. It should actively create an atmosphere and conditions for the innovation of water resource-related technologies, and achieve new breakthroughs in the development and secondary use of water resources, so as to provide technical support for the improvement of water use efficiency and the improvement of water resources–ecological management capacity nationwide.

The limitations of this study are appreciated. Firstly, although many scholars have used night light data as proxy variables for economic development, there have been objections in the academic field. Future research could further explore the rationality of this practice. Secondly, there are many ways to measure water resources–ecological management capacity. Future research can be carried out from other perspectives to further explore the water resources–ecological management capacity.

## Figures and Tables

**Table 1:** Water resources–ecological management index indicator system.

First Class Indicator	Second Class Indicator	Attribute	Weight
Water resources system	Total water resources	Positive	0.2441
	The amount of water supply	Positive	0.1652
Water social system	Per capita life water consumption	Negative	0.0241
	Per capita water consumption	Negative	0.0134
Water economic system	Proportion of agricultural water consumption	Negative	0.0515
	Proportion of industrial water consumption	Negative	0.0239
	Water consumption per RMB 10,000 of the GDP	Negative	0.0046
Water ecological environment	Per capita ecological water consumption	Positive	0.4021
	wastewater discharge per RMB 10,000 of the GDP	Negative	0.0911

**Table 2:** Classification of the coupling coordination level.

Stage of Development	Dysregulation Recession Stage			Transition Stage		Coordinated Development Stage		
	0–0.2	0.2–0.3	0.3–0.4	0.4–0.5	0.5–0.6	0.6–0.7	0.7–0.8	0.8–1
Coordination level	Severe imbalance	Moderate imbalance	Mild imbalance	Little imbalance	Basic Coordination	Moderate coordination	Good coordination	Quality Coordination

**Table 3:** SEM model estimation results.

Variables	Main	Spatial	Variance
PI	0.210 **		
TEC	0.003 **		
PGDP	0.032 ***		
FDI	0.001 **		
IS	−0.025 ***		
λ		0.422 ***	
sigma2_e			0.000 ***
Observations	527	527	527
Number of IDs	31	31	31

*** *p* < 0.01, ** *p* < 0.05.

## Data Availability

The data involved in the study can be obtained from the corresponding author upon reasonable request.
